# Brain mechanisms underlying the impact of attachment-related stress on social cognition

**DOI:** 10.3389/fnhum.2013.00816

**Published:** 2013-11-27

**Authors:** Tobias Nolte, Danielle Z. Bolling, Caitlin M. Hudac, Peter Fonagy, Linda Mayes, Kevin A. Pelphrey

**Affiliations:** ^1^Yale Child Study Center, Yale School of Medicine, Yale UniversityNew Haven, CT, USA; ^2^Research Department of Clinical, Educational and Health Psychology, Wellcome Trust Centre for Neuroimaging, University College LondonLondon, UK; ^3^Department of Psychology, University of Nebraska-LincolnLincoln, NE, USA

**Keywords:** mentalizing, social cognition, attachment, stress, Reading the Mind in the Eyes Test, bio-behavioral switch model

## Abstract

Mentalizing, in particular the successful attribution of complex mental states to others, is crucial for navigating social interactions. This ability is highly influenced by external factors within one's daily life, such as stress. We investigated the impact of stress on the brain basis of mentalization in adults. Using a novel modification of the Reading the Mind in the Eyes Test (RMET-R) we compared the differential effects of two personalized stress induction procedures: a general stress induction (GSI) and an attachment-related stress induction (ASI). Participants performed the RMET-R at baseline and after each of the two inductions. Baseline results replicated and extended previous findings regarding the neural correlates of the RMET-R. Additionally, we identified brain regions associated with making complex age judgments from the same stimuli. Results after stress exposure showed that the ASI condition resulted in reduced mentalization-related activation in the left posterior superior temporal sulcus (STS), left inferior frontal gyrus and left temporoparietal junction (TPJ). Moreover, the left middle frontal gyrus and left anterior insula showed greater functional connectivity to the left posterior STS after the ASI. Our findings indicate that attachment-related stress has a unique effect on the neural correlates of mentalization.

## Introduction

When we interact with others, we are interested not only in their visible features; vastly more important are the beliefs, desires, and intentions that lie behind their actions which must be inferred. Mentalizing refers to the human ability to perceive, represent, and reason about the intentions, beliefs, and psychological dispositions of one's self and others, e.g., appropriately attributing a mental state to a person's facially expressed sadness in order to infer their need to be comforted. The foundation for mentalizing is partly rooted in early infant–caregiver attachment relationships and matures over the course of development with continued interpersonal interactions (Fonagy et al., 2002, 2007; Luyten et al., under review). The neural system that underpins mentalizing comprises the medial prefrontal cortex (MPFC), temporoparietal junction (TPJ), temporal poles, superior temporal sulcus (STS), and posterior cingulate cortex (PCC)/precuneus (Gallagher and Frith, 2003; Saxe and Kanwisher, 2003; Frith and Frith, 2006; Lieberman, 2007). Additional work characterizing the neural correlates of mentalization has distinguished functional subnetworks within this system. Studies exploring neural correlates for implicit vs. explicit social cognition (Uddin et al., 2007; de Lange et al., 2008; Njomboro et al., 2008; Fonagy and Luyten, 2009; Pineda and Hecht, 2009; Rameson et al., 2010; for review) support a similar juxtaposition of implicit and explicit mentalizing operations. Indeed, implicit mentalization, defined as reflexive, automatic processing of mental states that does not require conscious, verbal efforts, engages the fusiform face area, STS, inferior frontal gyrus (IFG), and premotor areas (Allison et al., 2000; Pelphrey et al., 2003; Vuilleumier and Pourtois, 2007; Iacoboni, 2009; Vander Wyk et al., 2009). In contrast, explicit mentalization, defined as controlled, conscious, verbal efforts to decode one's mental states, involves the MPFC and TPJ (Amodio and Frith, 2006; Saxe, 2006).

Facial features, especially the eyes, convey a rich array of social information that plays a role in both implicit and explicit mentalizing processes (Pelphrey et al., 2002; Blair, 2003). In the Reading the Mind in the Eyes Test (RMET; Baron-Cohen et al., 2001), participants are asked to infer the mental states of others from static images depicting only the eye region. Mental state judgments are designed to evoke mentalizing operations in the participants. These judgments are usually compared with non-mentalizing tasks such as judging gender from the eyes. The RMET assesses aspects of both explicit and implicit mentalization, as it entails effortful and conscious decoding of intentional mental states as well as automatic processing of facial and emotionally salient stimulus components. As expected, the RMET reliably engages key nodes of the mentalizing system, including the MPFC, posterior STS, and IFG (Baron-Cohen et al., 1999; Russell et al., 2000; Adams et al., 2009; Moor et al., 2012).

Contemporary attachment theory describes attachment as a behavioral and physiological system that is biologically based and dynamically adapts to meet the needs of the individual's particular environment (Mikulincer and Shaver, 2007; Nolte et al., 2011). Perceived threats, fear and psychosocial stress have been shown to behaviorally activate an individual's attachment system, prompting a series of brain-modulated processes that ultimately aim to regulate the stress response by drawing on one's mental representations of attachment figures (i.e., parents, romantic partners) for comfort (Mikulincer and Shaver, 2007). As an example, when one experiences interpersonal stress that activates this attachment system, the capacity to understand someone else's mental state may be reduced, distorted, and less flexible, potentially corresponding to reduced attention to social cues (Luyten et al., under review). In line with this theory, there is growing evidence confirming that emotional arousal and psychosocial stress, especially when related to attachment relationships (with parents, romantic partners, close family members in situations entailing loss, abandonment, abuse etc.), behaviorally impair both explicit and implicit mentalizing (Repacholi and Slaughter, 2003; Moriguchi et al., 2006; Lieberman, 2007; Fonagy and Luyten, 2009). It remains unclear which specific characteristics of attachment-related stress underpin this interference (e.g., limited mentalizing resources due to increased allostatic load that can no longer be deployed to thinking about other people; (Nolte et al., 2011)). Moreover, based on Arnsten's (1998) dual process model, it has been proposed (Mayes, 2000, 2006) “that stress regulation involves the coordination between different neurochemical systems that serve as multilevel gates” for mentalizing processes (Luyten et al., submitted). Fonagy et al. (2011) have proposed that the relationship between stress, attachment activation and mentalization is best understood in terms of a *biobehavioral switch model*, which postulates a relative “switch from cortical to subcortical systems, from controlled to automatic (fast and inaccuracy-prone) mentalizing and subsequently to non-mentalizing modes” of social cognition under attachment-related stress. This is corroborated by more recent “default-interventionist dual-process theories” (Evans, 2011; Van Overwalle and Vandekerckhove, 2013) describing how implicit processing is viewed as a quick default solution to an evaluation, which may afterwards be modulated (accepted or corrected) by explicit reasoning. The latter, if not impaired by too much cognitive load (Spunt and Lieberman, 2013), is conceptualized to be a result of iterative reprocessing (Cunningham and Zelazo, 2007) via additional computation cycles passing information back and forth between lower and higher processing levels.

In this context, the impairment in mentalizing may simply reflect the impact of general autonomic arousal on cognitive performance in a broader range of executive functions or decoding processes, or it could signify a developmental origin of mentalizing within attachment relationships that is specifically affected during stress that involves attachment figures (Fonagy et al., 2007, 2011). Previous research supports the latter hypothesis, as associations have been reported between childhood parental maltreatment and impaired mentalization (Cicchetti et al., 2003; Pears and Fisher, 2005). Based on this and related evidence, it has been hypothesized that strong triggering of the attachment system may be associated with a relative deactivation of the blood oxygenation level-dependent (BOLD) response in brain regions underpinning the mentalizing system (Bartels and Zeki, 2000, 2004; Mayes, 2006; Satpute and Lieberman, 2006; Lieberman, 2007). The accuracy of mentalization inferences in everyday social cognition may thus be prone to modulation by the amount, but more importantly also the type of stress arousal in which they occur not the level of arousal. However, it is unclear whether stressors of different origins (i.e., general vs. socially-based vs. specifically attachment-related stress) impact mentalizing abilities differentially.

In this study we evaluated whether attachment-related stress, as a particular type of interpersonal stress, had a unique effect on mental state judgments compared with a general, non-interpersonal stressor. We expected that the different stressor types might also have differential affects on age judgments (the control task in the current study design), but that was not the main focus of our hypotheses.

Although behavioral studies employing the Trier Social Stress Test (TSST; Kirschbaum et al., 1993) have shown that stress affects subsequent performance on a variety of cognitive and physiological processes (Kudielka et al., 2004, 2007; Kuhlmann et al., 2005; Roelofs et al., 2005), the TSST was not shown to affect performance on the RMET (Smeets et al., 2009). However, The TSST does not operationalize stress in a truly individualized induction paradigm based upon individually significant interpersonal life events. Rather, it applies a standardized psychosocial stress protocol to each participant. Sinha (2009) developed a modified paradigm to evoke personalized, stressful arousal states in a laboratory setting. The paradigm specifically elicits idiosyncratic stress experiences for each participant. Using this technique, we previously provided evidence that exposure to an attachment-related stressor affects participants' accuracy scores on the RMET-R, compared with performance under no stress. After the stressor, accuracy increased in the gender-detection control task and decreased in the mentalization task. Increases in salivary cortisol and subjective ratings of experienced stress post-induction also supported the validity of the procedure (Nolte et al., submitted).

Here we sought to identify, via functional magnetic resonance imaging (fMRI) the brain mechanisms whereby attachment-related stress, relative to non-attachment-related stress, may differentially compromise mentalization. Participants completed a revised version of the RMET (RMET-R) and an age-judgment control task three times during an fMRI scan session: (1) at baseline, (2) after exposure to a general, non-interpersonal stress induction, and (3) after exposure to an attachment-related, interpersonal stressor. Based upon developmental (e.g., Cicchetti et al., 2003) and clinical research (Fonagy and Luyten, 2009), we hypothesized that re-experiencing an attachment-related, interpersonal (vs. a general, non-interpersonal) stressful life event would have a greater negative impact on the behavioral and neural correlates of mentalization, as indicated by reduced levels of activity within the nodes of the mentalizing network and altered patterns of functional connectivity among the nodes.

## Methods

### Participants

Eighteen healthy adult participants (nine male, all right-handed) were recruited via a graduate school volunteer system and completed this study. The majority of participants were undergraduate students; three worked as research assistants but were naïve to the study. The mean age was 20.9 years (*SD* = 1.44 years) and age ranged between 18 and 23 years. Participants were screened for MRI eligibility and against any psychiatric disorders using the Brief Symptoms Inventory (Derogatis, 1993). One participant was excluded for having head motion drift greater than 3 mm or degrees from position at the first volume acquisition during the functional scan. For one participant, the first block of the ASI condition was cut out due to an isolated period of motion. Two additional participants were excluded for failing to respond during the task for more than six consecutive trials (one full condition block). After these exclusions, 15 participants remained for analysis (eight male) with a mean age of 20.73 years (*SD* = 1.20), range 18–23 years. All participants gave written informed consent to participate in this protocol, which was approved by Yale University's Human Investigations Committee, and were compensated $50 for their participation.

### Experimental design

#### Stress inductions—creation of imagery scripts

The procedure was modified from an established paradigm tested in addiction and stress research (Sinha et al., 1999; Sinha, 2005; Chaplin et al., 2008). Two induction imagery scripts were created for each individual in a visit prior to scanning, with the aim of creating individually tailored, personally meaningful arousal states (Sinha et al., 1999; Sinha, 2005). Participants were asked to recall stressful events that had occurred to them during the past 12 months, both stressful events that were attachment-related (i.e., involving a significant other) and stressful events that were general (non-interpersonal) in nature. In this way, two types of inductions were created: attachment-related stress inductions (ASI) and general stress inductions (GSI). For ASI, participants were asked: “Please think of a stressful interpersonal situation with people very significant to you that left you feeling mad, sad, and upset and where you felt helpless and emotionally overwhelmed.” Common themes included relationships ending, attending a relative's funeral or having to move out of the parental home. These specific situations, according to attachment theory (e.g., Bowlby, 1982; George and West, 2001) are not only social in nature but are additionally characterized by the fact that the involved other is relevant and salient to set the individual's attachment system in motion. If a provided narrative did not fit these criteria further probing was used until appropriate material was given. For GSI, participants were asked: “Please think of a stressful situation that left you feeling mad, sad, and upset and where you felt helpless and emotionally overwhelmed that did not involve another person significant to you.” Common themes here included the crucial period of preparation for final exams, the loss of an important object such as a wallet with all its contents, or missing a flight.^1^

All participants rated their perceived stress on a 10-point Likert scale where scores from 1 (*not at all stressful*) to 10 (*the most stress they had felt recently in their life*) were applied to evaluate the provided vignette (Wolpe, 1969). For both induction types, only situations rated 8 or above on the perceived stress scale were accepted as appropriate for script development. In order to enhance the re-experience of their selected events and to render scripts as ecologically valid as possible, participants also chose from a list of physical reactions to stress that reflected their bodily sensations at the time of the event (e.g., “There is a sinking feeling in my chest”). Scripts were then edited following a semi-standardized procedure (Sinha, 2005) and recorded by the same female person, yielding personalized induction imagery scripts of about 5 min in length. Scores for subjective stress experience (Wolpe, 1969) were obtained again at the beginning of the debriefing after scanning and showed no difference in mean scores when comparing both stress conditions in a paired samples *t*-test [*t*_(14)_ = 1.07, *p* = 0.34].

#### Measurement of social cognition

**Figure A1:**
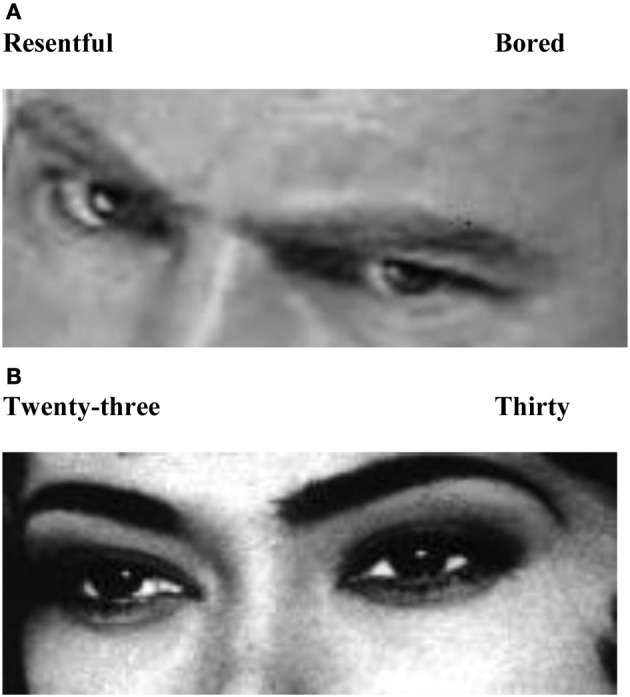
**Examples of single trials. (A)** emotion recognition task; **(B)** control task, age judgments.

The experimental task was based on the RMET (Baron-Cohen et al., 2001), a task in which participants are asked to match static grayscale images of emotionally expressive human eyes to a corresponding mental state label. In this study, participants performed a revised version of this task (RMET-R) whereby the original gender-attribution control task was replaced by judgments about displayed age (Figure A1). This modification was implemented in order to increase the difficulty of the control task, rendering it more comparable to the experimental task. Further, to allow for stimulus processing within the desired presentation time, only two out of the original four labels were retained (one being the target, the other one a foil), and age choices were written as words rather than numerals to ensure lexical similarity. Additional stimuli (Nolte et al., submitted) were created to obtain a sufficient trial number (72 images in total). The experiment comprised the collection of three functional runs all within the same scan session (baseline, post-GSI, post-ASI), referred to as conditions. In each condition blocks alternated between mental state and age judgment tasks (four blocks of each task, eight blocks in total) with a 12 s fixation cross between blocks. The total duration per condition was 4.6 min. In each block, six trials were performed in which stimuli were shown for 3500 ms followed by 500 ms of fixation after each stimulus presentation. In each condition, different stimuli were shown in order to minimize potential practice effects due to repeated exposure and each stimulus had to be judged twice, once for age, once for mental state.

Both RMET-R stimuli and auditory inductions (during which participants were asked to close their eyes) were presented using E-Prime 2.0 software (Psychological Software Tools, Inc., Pittsburgh, PA). All participants completed three functional runs (conditions) of the RMET-R task: an initial baseline measurement, which was used to identify the neural network subserving RMET-R-elicited mentalization, one following the GSI, and one following the ASI. The presentation order of induction type was balanced between participants in order to control for carry-over effects so that half of them re-experienced a GSI first and the other half re-experienced an ASI first.

### MRI data acquisition

Scanning was performed on a Siemens 3T TIM Trio scanner (Siemens, Erlangen, Germany) at the Magnetic Resonance Research Center, Yale University School of Medicine. T1-weighted anatomical images were acquired using an MPRAGE sequence (TR = 2530 ms; TE = 3.34 ms; FOV = 25.6 cm; image matrix = 64^2^; 1 × 1 × 1 mm). For 11 participants, in each RMET-R run, 156 whole-brain functional images were acquired using a single-shot, gradient-recalled echo planar pulse sequence (TR = 2000 ms; TE = 25 ms; flip angle = 60°; FOV = 22 cm; image matrix = 64^2^; voxel size = 3.2 × 3.2 × 3.2 mm; 34 slices) sensitive to BOLD contrast. For the remaining four participants, each RMET-R run consisted of 154 whole-brain functional images acquired in the same manner. This two-volume discrepancy was due to a scanner operator inconsistency, and did not overlap with data acquisition during the presentation of task stimuli.

### Data analysis

#### Behavior

For 13 of the 18 participants (including those removed from fMRI analyses), behavioral data including percentage of correct mental state and age judgment responses and mental state and age judgment reaction times were recorded during the scan. The data from the two participants who failed to respond to six or more consecutive trials were not included in behavioral analyses. In addition, behavioral data from three participants were lost to software errors. Age-judgment accuracy was calculated on the basis of norm data from an external population of 13 students, age range 22–31 years (*SD* = 2.61). The cut-off for determining correct responses was 60% consensus between participants. For 12 out of the 72 stimuli no such consensus was established and those stimuli were excluded from accuracy and reaction time analyses. We performed four within-participant repeated measures ANOVAs comparing the three task conditions (baseline, post-GSI, post-ASI) for differences in both correct responses and reaction times, for mental state and age judgments separately. *Post-hoc* paired samples *t*-tests were performed to compare mean accuracy and average reaction times for mental state judgments between each condition.

#### MRI

Data were analyzed with Brain Voyager QX version 2.0.8 (Brain Innovation, Maastricht, Netherlands). The three volumes prior to the onset of the first stimulus event were discarded to allow for T1 equilibrium. Preprocessing of the functional data included interleaved slice time correction using cubic spline interpolation, three-dimensional motion correction using trilinear/sinc interpolation, linear trend removal, temporal smoothing with a Gaussian FWHM of 2.8 s, and temporal high-pass filtering with Fourier basis set, using three cycles per time course. Upon examination of estimated motion plots and cine loops, one participant was excluded due to greater than 3.0 mm (or degrees) deviation or rotation from the initial estimated center of mass in any direction. Functional datasets were co-registered to within-session anatomical images, which were in turn normalized to Talairach space. On a single-participant level, general linear model (GLM) analyses were performed by defining two task predictors as boxcar functions with values of 1 during experimental blocks (mental state or age) and 0 otherwise, and convolving each of these task predictors with a double-gamma hemodynamic response function. The two task predictors, as well as motion predictors depicting movement in each of the three translations and three rotations were included in each single-participant GLM, with the task functions as the only predictors of interest. For each participant, three distinct GLM analyses were performed on the functional data collected during the RMET-R: baseline, post-GSI, and post-ASI.

To identify the network of regions subserving mentalization during the RMET-R, a multi-participant random-effects GLM analysis was performed on functional data obtained at baseline (with no prior stressor) by comparing activation during mental state judgments to activation during age judgments. Patterns of differential activation were identified at a voxel-wise false discovery rate (Genovese et al., 2002) threshold of *q* < 0.05 with only clusters of four or more contiguous functional voxels displayed.

A group-level activation mask was created from the initial baseline run to investigate stressor-related differences in mentalization activation specifically within regions modulated by the RMET-R. A region-of-interest mask was created from regions differentially active in mental state vs. age judgments in the baseline RMET-R run at the threshold *q* < 0.05. Subsequently, activation in each of the two judgment conditions in the RMET-R post-stressor (GSI and ASI) was assessed by performing two multi-participant random-effects GLM analyses (one per stressor) using the mask created from the task analysis at baseline. Whole-brain volume maps of activation were created for each participant for the mental state and age judgment conditions separately in each of the post-stressor RMET-R runs. Within each judgment type, activation during the RMET-R post-GSI was subtracted from activation during the RMET-R post-ASI. This subtraction was done for each participant and the differences were compared to zero, constituting a voxel-wise paired-sample *t*-test. This allowed us to look at task-related regions that were differentially activated depending on the stressor (GSI or ASI) in mental state and age judgments separately. These results were assessed at a statistical threshold of *p* < 0.05, with a cluster threshold of four functional voxels, as the number of voxel-wise comparisons being made was already restricted by the functional mask. The functional mask left only voxels for which our hypothesis predicted modulation by stress induction. For this reason, we chose a very liberal cluster threshold to explore all possible stress modulations. Alternatively, when we used a cluster threshold estimator based on 1000 Monte-Carlo simulations, 13 voxels was estimated to correspond to an α < 0.05 for the stressor comparisons of both age and mental state judgments. This calculated cluster threshold only eliminates the significance of the result in left IFG.

Furthermore, a modified psychophysiological interaction (PPI) analysis (Friston et al., 1997) was performed on each RMET-R dataset to determine regions of greater functional connectivity in mental state vs. age judgments to a functionally-defined region of the left posterior STS that showed greater activation to mental state (vs. age) judgments in the current study (peak coordinate −51, −34, 1; 3674 1 mm^3^ voxels). Prior to the connectivity analyses, the global mean (average signal across voxels) was removed from each volume as a surrogate method for physiological artifact removal (Fox et al., 2005). A random-effects whole-brain GLM analysis identified a left posterior STS region (136 functional voxels) with greater activation to mental state vs. age judgments during the baseline RMET-R. Using this functionally defined seed region, PPI regressors for each judgment type were created separately by multiplying the hemodynamic response function-convolved task regressor for each judgment type by the preprocessed and normalized left posterior STS time course for each participant. This PPI function along with the task predictors and left posterior STS time course were used as regressors in a multi-participant random-effects GLM analysis in each RMET-R run for each judgment type. The PPI function was used as the only predictor of interest. For each of the two post-stressor RMET-R runs, whole-brain volume maps were created for each participant, modeling the PPI predictor for both mental state judgments and age judgments separately. As described in the functional activation analysis, a whole-brain voxel-wise paired *t*-test was performed to contrast differential functional connectivity post-GSI vs. post-ASI for each type of judgment (age and mental state). This contrast allowed us to define regions that showed greater functional connectivity to the left posterior STS in the task post-ASI compared with post-GSI. This comparison of functional connectivity was made separately for mental state judgments and age judgments. The two contrasts were assessed at a threshold of *p* < 0.05, corrected for multiple comparisons with a cluster threshold of 34 functional voxels (Forman et al., 1995; Xiong et al., 1995). This cluster threshold was determined using the Brain Voyager QX Cluster-level Statistical Threshold Estimator plugin to correspond to a whole-brain corrected threshold of α < 0.05. After 1000 iterations of a Monte-Carlo simulation, an alpha value is assigned to each cluster size based on its relative frequency.

## Results

### Behavior

The mean percentages of correct responses and reaction times in each task are displayed in Table 1. Mean percentages of correct responses [*t*_(12)_ = 0.21, *p* = 0.84] and reaction times [*t*_(12)_ = 1.89, *p* = 0.08] comparing mental state and age judgments at baseline did not differ significantly.

**Table 1:** **Within-participant repeated measures ANOVAs comparing three task conditions for differences in correct responses and reaction times**.

**Variable**	**Baseline mean (*SD*)**	**Post-GSI mean (*SD*)**	**Post-ASI mean (*SD*)**	***F*_(2, 24)_**	***P***	
MS CR	74.70 (6.05)	74.98 (8.60)	67.03 (10.00)	3.59	0.04
MS RT	2187 (289)	2075 (283)	2008 (220)	10.13	0.01
Age CR	73.85 (11.57)	74.62 (14.06)	73.85 (11.39)	0.04	0.97
Age RT	2102 (244)	2038 (215)	1998 (317)	1.28	0.20

Abbreviations: MS, mental state judgments; age, age judgments; CR, percentage of correct responses; RT, reaction times in milliseconds; GSI, general stress induction; ASI, attachment stress induction. Post-hoc paired samples comparisons for CR in MS: significant differences in CR between baseline and post-ASI [*t*_*(12)*_ = 2.30, p < 0.05] and between post-GSI and post-ASI [*t*_*(12)*_ = 2.20, p < 0.05] with highest CR at baseline and lowest post-ASI. Post-hoc t-tests for RT during MS: significant differences between baseline and post-GSI [*t*_*(12)*_ = 4.12, p < 0.01] and between baseline and post-ASI [*t*_*(12)*_ = 3.65, p < 0.01] with longest reaction times at baseline and RT times post-ASI.

Four within-participants repeated measures ANOVAs comparing the three task conditions for differences in both correct responses and reaction times were performed for mental state and age judgments separately (see Table 1). For age judgments, this analysis did not yield significant differences for either correct responses or reaction times. Results of the ANOVA for mental state judgments indicated significant differences between task conditions for correct responses (*F* = 3.59, *p* = 0.04) and reaction times [*F*_(2, 24)_ = 10.13, *p* = 0.01].

*Post-hoc* paired samples comparisons for correct responses in mental state judgments indicated that there were significant differences in the percentage of correct responses between baseline and post-ASI [*t*_(12)_ = 2.30, *p* < 0.05] and between post-GSI and post-ASI [*t*_(12)_ = 2.20, *p* < 0.05] with highest accuracy at baseline and lowest post-ASI. *Post-hoc t*-tests for reaction times during mental state judgments revealed significant differences between baseline and post-GSI [*t*_(12)_ = 4.12, *p* < 0.01] and between baseline and post-ASI [*t*_(12)_ = 3.65, *p* < 0.01] with longest reaction times at baseline and shortest reaction times post-ASI.

### fMRI

**Figure 1:**
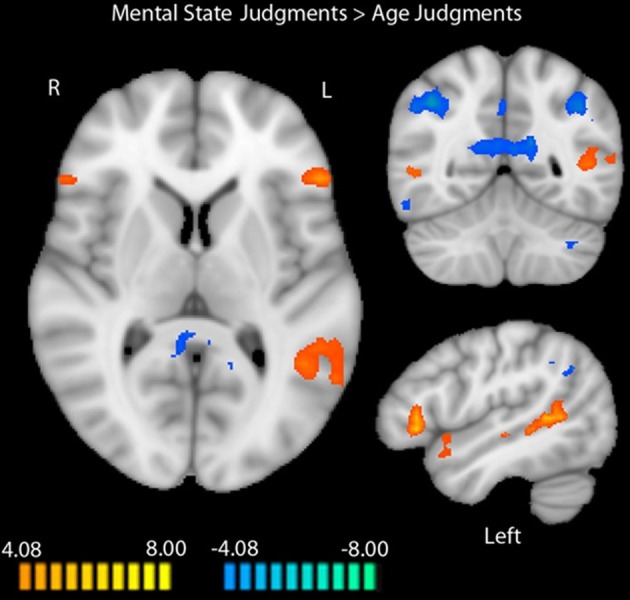
**Regions differentially activated by mental state and age judgments in the RMET-R administered with no previous stress induction**. Regions in orange showed more activation to mental state judgments, while regions in blue showed more activation to age judgments (random-effects GLM, *q* < 0.05, *k* = 4). Data is interpolated to 1 mm^3^ resolution for presentation.

We identified a network of brain regions that responds differentially to age and mental state judgments during the RMET-R in the absence of any pre-task stress induction (Figure 1). Peak coordinates, effect-size values, size, and anatomical labels for the regions of differential activation between the two judgment types are displayed in Table 2.

**Table 2:** **Activation in RMET-R at baseline**.

	***X***	***Y***	***Z***	**Size**	***t***	***p***
**MENTAL STATE > AGE JUDGMENTS**						
Right pSTS	54	−31	1	156	5.43	<0.001
Right IFG	51	26	1	388	6.75	<0.001
Left posterior insula	−39	−43	19	134	7.16	<0.001
Right MTG	48	−52	1	177	5.73	<0.001
Left STG	−51	8	−11	849	8.18	<0.001
Left mSTS	−54	−16	−2	192	6.55	<0.001
Left pSTS	−51	−34	1	3674	8.56	<0.001
Left parahippocampal gyrus	−42	−16	−14	203	5.85	<0.001
Left IFG	−51	26	7	1410	10.04	<0.001
DMPFC	−9	−4	55	517	6.51	<0.001
**AGE > MENTAL STATE JUDGMENTS**						
Right IPL	39	−49	40	9259	−11.76	<0.001
Right MFG	39	29	19	240	−5.92	<0.001
PCC/precuneus	6	−34	31	13822	−10.14	<0.001
Right DLPFC	30	5	46	2297	−6.19	<0.001
Right ITG	60	−37	−11	1753	−9.72	<0.001
Left IPL	−39	−55	43	4153	−7.73	<0.001
Left cerebellum	−33	−55	−35	491	−8.39	<0.001

Regions identified in a whole-brain contrast of mental state and age judgments. Talairach coordinates and statistics refer to the voxel with the maximum signal change in each region of interest. Abbreviations: pSTS, posterior superior temporal sulcus; IFG, inferior frontal gyrus; MTG, middle temporal gyrus; STG, superior temporal gyrus; mSTS, middle superior temporal sulcus; DMPFC, dorsomedial prefrontal cortex; IPL, inferior parietal lobule; MFG, middle frontal gyrus; DLPFC, dorsolateral prefrontal cortex; ITG, inferior temporal gyrus.

As illustrated in Figure 1, regions that showed increased activation during mental state judgments at baseline included bilateral IFG (right: *t* = 6.75, *p* < 0.0001; left: *t* = 10.04, *p* < 0.0001), right posterior STS (*t* = 5.43, *p* < 0.0001), left posterior insula (*t* = 7.16, *p* < 0.0001), right middle temporal gyrus (*t* = 5.73, *p* < 0.0001), left superior temporal gyrus (*t* = 8.18, *p* < 0.0001), and left middle and posterior STS (middle: *t* = 6.55, *p* < 0.0001; posterior: *t* = 8.56, *p* < 0.0001). Additional regions that were more active during mental state judgments included the left parahippocampal gyrus (*t* = 5.85, *p* < 0.0001) and dorsoMPFC (*t* = 6.51, *p* < 0.0001).

Regions that showed more activation to age judgments included bilateral inferior parietal lobules (right: *t* = −11.76, *p* < 0.0001; left: *t* = −7.73, *p* < 0.0001), right middle frontal gyrus (MFG; *t* = −5.92, *p* < 0.0001), PCC extending into precuneus (*t* = −10.14, *p* < 0.0001), right dorsolateral prefrontal cortex (*t* = −6.19, *p* < 0.0001), right inferior temporal gyrus (*t* = −9.72, *p* < 0.0001), and left cerebellum (*t* = −8.39, *p* < 0.0001).

When we compared activation during mental state judgments between the two types of stress inductions in the identified task-related areas, we localized regions that were more active during mental state judgments after the GSI compared with the ASI. These regions included the left IFG (*t* = −2.93, *p* < 0.05), left posterior STS (*t* = −5.02, *p* < 0.001), and left temporal parietal junction (*t* = −4.83, *p* < 0.001). Thus, post-ASI, activation in these three regions decreased significantly in comparison to post-GSI. There were no regions that were more active during mental state judgments after the ASI compared with the GSI.

**Figure 2:**
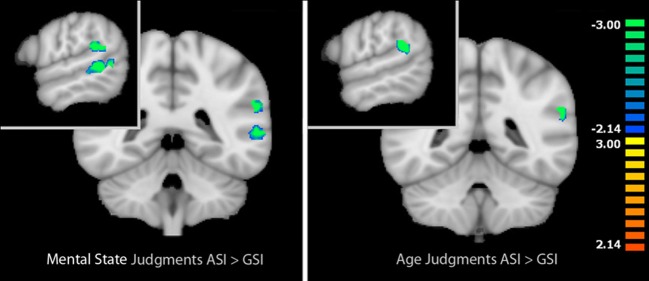
**Left: Regions that showed differential activation between mental state and age judgments in the baseline RMET-R that were modulated by stress induction type during mental state judgments**. Right: Regions that showed differential activation between mental state and age judgments in the baseline RMET-R that were modulated by stress induction type during age judgments. Regions in blue reflect decreased activation post-ASI compared with post-GSI (masked voxel-wise paired *t*-test, *p* < 0.05, *k* = 4). Data is interpolated to 1 mm^3^ resolution for presentation.

The same analysis within age judgments identified only one region that was more active during age judgments after the GSI compared with the ASI: the left TPJ (*t* = −5.08, *p* < 0.001). Again, no regions were more active during age judgments after the ASI compared with the GSI. Results of the comparison between stress inductions within age and mental state judgments are displayed in Figure 2. Peak coordinates, effect-size values, size, and anatomical labels for the regions of differential activation between the two stress inductions are displayed in Table 3.

**Table 3:** **Activation during mental state judgments and age judgments post-ASI vs. post-GSI**.

	***X***	***Y***	***Z***	**Size**	***t***	***p***
**MENTAL STATE JUDGMENTS**						
Left IFG	−45	29	11	137	−2.93	0.011^*^
Left pSTS	−54	−46	3	1539	−5.02	<0.001
Left TPJ	−57	−40	22	490	−4.83	<0.001
**AGE JUDGMENTS**						
Left TPJ	−56	−43	22	359	−5.08	<0.001

Regions identified in a paired t-test (within voxels showing differential activation to mental state and age judgments during the RMET-R at baseline) to show differential activation post-ASI vs. post-GSI for each judgment type (mental state and age).

* Left IFG did not survive cluster correction to α < 0.05. Abbreviations: IFG, inferior frontal gyrus; pSTS, posterior superior temporal sulcus; TPJ, temporoparietal junction.

When we compared functional connectivity to the functionally-defined left posterior STS during mental state judgments after the two stress inductions, regions that showed greater functional connectivity to the left posterior STS after the ASI included the left MFG (*t* = 4.90, *p* < 0.001) and left anterior insula (AI; *t* = 5.24, *p* < 0.001). No regions were more functionally connected to the left posterior STS during mental state judgments post-GSI.

**Figure 3:**
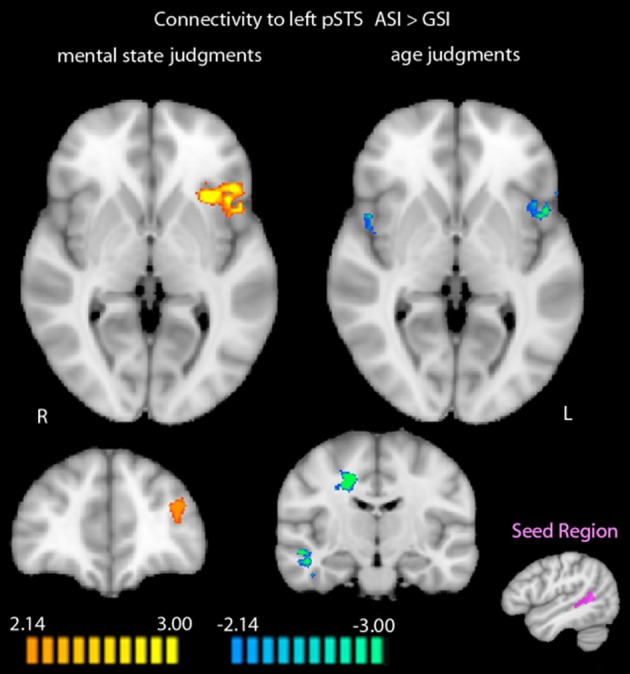
**Differential functional connectivity to the left posterior STS during mental state and age judgments after the ASI vs. the GSI**. Regions in orange showed greater functional connectivity to the left posterior STS during judgments post-ASI. Regions in blue showed greater functional connectivity to the left posterior STS during judgments post-GSI (*p* < 0.05, *k* = 34). The seed region for the connectivity analysis (shown in purple) was defined by greater activation to mental state judgments compared with age judgments in the RMET-R at baseline. Data is interpolated to 1 mm^3^ resolution for presentation.

Exploring differential connectivity to the left posterior STS during age judgments revealed several regions that differed between the two stress inductions. The cuneus showed increased functional connectivity to the left posterior STS following the ASI compared with the GSI (*t* = 3.78, *p* < 0.01). Bilateral AI (right: *t* = −3.94, *p* < 0.01; left: *t* = −5.66, *p* < 0.001), right middle temporal gyrus extending into parahippocampal gyrus and fusiform gyrus (*t* = −5.37, *p* < 0.001), right dorsal anterior cingulate cortex (*t* = −6.28, *p* < 0.001), and left fusiform gyrus (*t* = −4.58, *p* < 0.001) were more functionally connected to the left posterior STS during age judgments post-GSI vs. post-ASI. Results of the two connectivity analyses are displayed in Figure 3. Peak coordinates, effect-size values, size, and anatomical labels for the regions of differential connectivity between the two stress inductions are displayed in Table 4.

**Table 4:** **Differential functional connectivity during mental state and age judgments post-ASI vs. post-GSI, using a seed region of the left posterior STS defined by differential activation to mental state and age judgments in the baseline RMET-R**.

	***X***	***Y***	***Z***	**Size**	***t***	***p***
**MENTAL STATE JUDGMENTS**						
**ASI > GSI**						
Left anterior insula	−24	14	−8	6495	5.24	<0.001
Left MFG	−36	41	25	939	4.90	<0.001
**AGE JUDGMENTS**						
**ASI > GSI**						
Cuneus	9	−79	43	942	3.78	0.0020
**GSI > ASI**						
Right anterior insula	48	8	−2	980	−3.94	0.0015
Left anterior insula	−48	11	10	1042	−5.66	<0.001
Right MTG/PHG/FFG	42	−28	−8	5679	−5.37	<0.001
Right dorsal ACC	21	−13	37	1068	−6.28	<0.001
Left FFG	−39	−43	−23	928	−4.58	<0.001

Regions identified in a whole-brain voxel-wise paired t-test to show differential functional connectivity to left posterior STS post-ASI vs. post-GSI for each judgment type (mental state and age). Abbreviations: MFG, middle frontal gyrus; MTG, middle temporal gyrus; PHG, parahippocampal gyrus; FFG, fusiform gyrus; ACC, anterior cingulate cortex.

## Discussion

Our results provide three important contributions. First, we replicated fMRI findings with the RMET using a more complex control task. Second, we identified differences in stress-induced modulation of brain activation during age and mental state judgments based on the nature of the stressor (attachment-related vs. general). Third, and importantly, we demonstrated that interpersonal contextual factors, specifically the activation of an attachment-specific stress system (compared with a more general stress response system), uniquely modulates the functioning of brain mechanisms involved in mentalizing. Each of these aspects will be discussed below as well as limitations of the study.

### Neural correlates of the revised RMET

The results obtained from the RMET-R at baseline replicated findings from previous studies on the neural processes underpinning mental state decoding operationalized with the RMET (Baron-Cohen et al., 1999; Russell et al., 2000; Adams et al., 2009). Regions previously implicated in mentalization that showed increased activation during mental state judgments in the present study included bilateral IFG, right posterior STS, right middle temporal gyrus, left superior temporal gyrus, and left middle and posterior STS. Additional regions found to be more active during mental state judgments included the left posterior insula, left parahippocampal gyrus and dorsoMPFC. Overall, these results suggest that when performing our extended version of the revised RMET with a modified control task, the previously established mentalization neural network is engaged.

### Age judgments

The control task allowed the delineation of a network of brain areas involved in making age judgments. To date, this important component of social information processing using neuroimaging has been investigated with relatively few tasks (e.g., Harris and Fiske, 2007; Winston et al., 2007; Moor et al., 2012). We asked participants to choose between two age attributions (but, for consistency, displayed the ages as words rather than numbers) in a task similar to the RMET-R. Regions activated in response to age judgments as presented in this study included bilateral intraparietal lobules extending into the intraparietal sulcus, as well as the right MFG, PCC extending into the precuneus, right dorsolateral prefrontal cortex, right inferior temporal gyrus, and left cerebellum. Previous findings on numerical processing (Dehaene et al., 2003; Cantlon et al., 2006; Cohen Kadosh et al., 2007; Santens et al., 2009) have highlighted the role of the intraparietal sulcus for both non-symbolic numerical abilities and symbolic numerical processing.

### Attachment-related stress and mentalizing

We hypothesized a differential effect of stress induction type (ASI vs. GSI) on behavioral performance and on the neural system for mentalizing. In line with our main prediction, we found significantly reduced accuracy scores and reduced reaction times, specifically after ASI. This decrease is unlikely to be attributable to fatigue as we did not find order effects for the stress types.

Moreover, we found reduced activity in three brain regions during the mental state task after experiencing the stressor that contained attachment-related life events. Attachment-related stress compared with a general stress resulted in differential activity during mental state decoding in the left posterior STS, TPJ, and IFG. In contrast, there was a significant difference between the effects of stress induction types on modulation of activity during age judgments only in the left TPJ. Additionally, differential functional connectivity to the left posterior STS during both tasks post-ASI, compared with post-GSI, corroborated the hypothesis that attachment-related stress uniquely alters the engagement of brain networks involved in mentalizing.

The results confirmed that re-experiencing attachment-related stress specifically interferes with both behavioral performance and with the functioning of brain regions associated with mentalizing in a sample of healthy young adults. This provides support for Fonagy and Luyten's model (Fonagy and Luyten, 2009; Nolte et al., 2011; Luyten et al., under review), which proposes that stress-induced affective states compromise social cognition in the context of intimate relationships. Indeed, reduced accuracy and the reduction in activation of areas underpinning mentalization might be related to the setting in motion of the behavioral attachment system (Mikulincer and Shaver, 2007). Our findings lend preliminary support to the *biobehavioral switch model* Fonagy et al. (2011) as participants make post-ASI judgments that are faster but at the cost of more errors which is in line with reflexive, “jumping to conclusion” responses. Future research may address what direction the reduced accuracy in the RMET task takes, e.g., a shift toward more error-prone inference regarding negative or less ambiguous stimuli.

On a neural level, our findings are, furthermore, indicative of a substantial reduction in activation during mental state judgments, due to attachment-related stress. This reduction in activation occurred in several regions of the core neural system that integrates controlled, effortful (explicit) and automatic, reflexive (implicit) mentalization (the TPJ, IFG, and STS). This calls for further investigations of the role of contextual manipulations of basic social cognitive processes as the neural mechanisms of iterative reprocessing and the computational rendering of implicit to explicit processing modes remain unclear (Spunt and Lieberman, 2013; Van Overwalle and Vandekerckhove, 2013). Attachment-related stress also significantly modulated activation during age judgments, but only in one region of the mentalizing network we identified (TPJ), and without significant behavioral changes.

Our identification of differential effects of attachment-related and general stress on activation of the mentalization network in mental state judgments is complemented by PPI analyses showing different patterns of functional connectivity to the left posterior STS in each of these conditions. Specifically, a dissociable pattern of functional connectivity was found in the AI. During mental state judgments, functional connectivity between the left posterior STS and left AI increased post-ASI compared with post-GSI. In contrast, during age judgments, functional connectivity between the left posterior STS and bilateral AI decreased post-ASI compared with post-GSI. The AI has been associated with subjective awareness (Craig, 2009) and emotion processing (Critchley et al., 2005; Hennenlotter et al., 2005; Jabbi et al., 2007), and has been suggested to hold a key role in empathy (for review, see Singer et al., 2009). Further, along with the MFG (which was also found in the present study to show increased functional connectivity with the left posterior STS during mental state judgments post-ASI), AI has been shown to be preferentially active in difficult vs. easy moral judgments (Greene et al., 2004). While our aforementioned results showed that left posterior STS activation is compromised following the attachment-related stressor, increased temporal coupling with the AI and MFG post-ASI is in line with decreased performance accuracy after experiencing attachment-related stress. In contrast, decreased temporal coupling of the left posterior STS and AI during age judgments post-ASI supports the specificity of this hypothesis to mentalization processes. Taken together with the decrease in functional connectivity between the left posterior STS and bilateral fusiform gyrus during age judgments post-ASI, the identified pattern of functional connectivity to the left posterior STS during age judgments post-ASI (compared with post-GSI) implies a dissociation of the left posterior STS from brain regions subserving both lower level face processing (fusiform gyrus) and higher level empathic processes (AI), potentially reflecting a shift toward non-mentalization as hypothesized within the *biobehavioral switch model*.

### Limitations

Several limitations in the current study have to be taken into account. First, for a non-clinical sample, 15 participants may be considered a relatively small sample size. Further, behavioral analyses were limited to only 13 participants, 12 of which overlapped with the fMRI group. While we are confident in our interpretations of the presented data, the small number of participants with fMRI and behavioral data (12) occluded the possibility of performing direct investigations of correlations between behavioral responses and fMRI data. A larger sample size would also allow for the investigation of gender differences in the observed neural processes. Although our results do not provide direct evidence regarding a link between neural and behavioral findings, previous work has established reliable relationships between the behavioral performance of the RMET and its neural correlates (Baron-Cohen et al., 1999; Russell et al., 2000; Adams et al., 2009; Moor et al., 2012). Moreover, limitations related to the age task should be addressed in future studies by using a task for which the difficulty or cognitive load between task and control task can be quantified better.

Second, there are limitations due to the lack of diversity in the specific sample investigated. Future research should assess the neural processes at the focus of the current study in populations more ethnically and developmentally diverse to allow for generalizability. Finally, even though we were specifically interested in the subjective experience of distress, we do not provide evidence for alterations of psychophysiological indices such as heart rate variability or cortisol levels associated with the different stress conditions. These may enhance the understanding of the differential effect of ASI and GSI on neural processes. While we took efforts to match the inductions on the level of induced stress, we do not have data that speak to the simultaneous induction of other emotions and underpinning neural networks that may have occurred with more intensity during ASI (such as fear, anger, sadness, or a complex combination thereof). However, the ability of attachment-related stressors to induce other emotions is not a confound, but a potential means by which this particular type of stressor induces its unique effects on cognition and mentalization. Whilst theory-driven, the current study compared attachment-related stress as a specific case of interpersonal stress with a more GSI. Although there is some evidence from behavioral studies that suggests otherwise, future studies need to investigate whether other social stressors are salient enough to elicit compromised mentalizing performance (a general social script effect) or whether this is uniquely due to the attachment aspect. With the current design it is also undecided whether the behavioral effects (faster and less accurate) are specifically related to the negative social scripts or whether positive social scripts might have yielded similar priming responses. Furthermore, future studies will have to disentangle whether the differential stress effects reported are more generally due to the difference between their social vs. non-social nature and whether individual differences in attachment may moderate the impairment in mentalizing (e.g., Vrtička and Vuilleumier, 2012).

### Conclusion

In sum, using a novel modification of the RMET, we identified the left posterior STS and left IFG as brain regions that were modulated by attachment stressors during mental state judgments accompanying compromised behavioral performance, as well as the left TPJ, which showed an effect of stressor type during both mental state and age judgments. This finding follows behavioral work showing the specific effects of attachment-related stress on mentalizing, and thus suggests a neurobiological basis by which these effects likely occur. This development is important for the future elucidation of transient and interpersonal factors that can influence behavioral and neural correlates of social cognition in healthy populations, and particularly in relation to psychopathologies with impaired mentalizing. In addition, these results provide a more detailed understanding of the effects of stress on social cognition, suggesting that factors including the nature of the stressor and the type of social reasoning interact in meaningful ways to shape neural correlates of social cognition.

### Conflict of interest statement

The authors declare that the research was conducted in the absence of any commercial or financial relationships that could be construed as a potential conflict of interest.

## Footnotes

^1^By implication, in most cases other people were referenced in these scripts so that they were not strictly non-social but stories were excluded if the other persons could be viewed as significant others to the participant in line with attachment theory.
